# Supplementary material for: Forecasting the COVID-19 transmission in Italy based on the minimum spanning tree of dynamic region network
Source: PeerJ. 2021 Jun 29;9:e11603. doi: 10.7717/peerj.11603 (PMC8253113; doi:10.7717/peerj.11603)
Supplement: Supplemental Information 2 [file peerj-09-11603-s002.docx]

**Forecasting the COVID-19 transmission in Italy based on the minimum spanning tree of dynamic region network**

## Min Dong ^1^, Xuhang Zhang ^1^, Kun Yang ^1^, Rui Liu ^2, 3*^, Pei Chen ^2*^

## Supplementary Table

**Table S1:** The adjacent information of 21 regions/autonomous province in Italy.

| Node | Regions/Autonomous provinces | Adjacent information |
| --- | --- | --- |
| 1 | Abruzzo | 8, 11, 12 |
| 2 | Basilicata | 4, 5, 14 |
| 3 | P.A. Bolzano | 18, 21 |
| 4 | Calabria | 2, 16 |
| 5 | Campania | 2, 8, 12, 14 |
| 6 | Emilia Romagna | 9, 10, 11, 13, 17, 21 |
| 7 | Friuli Venezia Giulia | 21 |
| 8 | Lazio | 1, 5, 11, 12, 17, 19 |
| 9 | Liguria | 6, 13, 17 |
| 10 | Lombadia | 6, 13, 18, 21 |
| 11 | Marche | 1, 6, 8, 17, 19 |
| 12 | Molise | 1, 5, 8, 14 |
| 13 | Piemonte | 6, 9, 10, 20 |
| 14 | Puglia | 2, 5, 12 |
| 15 | Sardegna | - |
| 16 | Sicilia | 4 |
| 17 | Toscana | 6, 8, 9, 11, 19 |
| 18 | P.A. Trento | 3, 10, 21 |
| 19 | Umbria | 8, 11, 17 |
| 20 | Valle d'Aosta | 13 |
| 21 | Veneto | 3, 6, 7, 10, 18 |

## Supplementary Figures


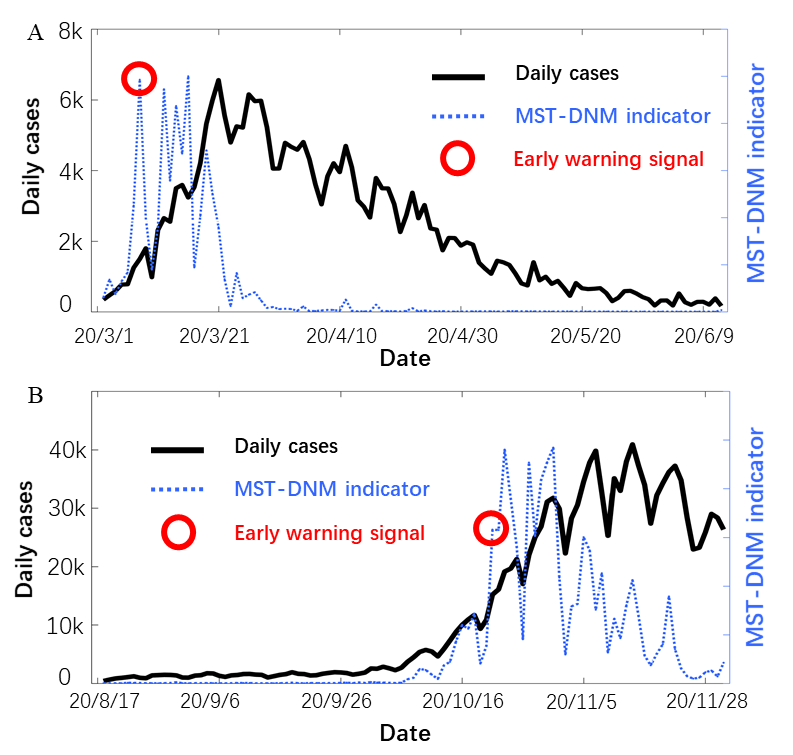


**Figure S1: Forecast of the COVID-19 outbreaks in Italy with the model including Sardinia.** In each subgraph, the left y-axis is the number of daily cases in in all regions of northern Italy and the right y-axis is the MST-DNM indicator of corresponding date. (A) Forecasting and warning of the first wave of COVID-19 outbreak in northern Italy. (B) Forecasting and warning of the second wave of COVID-19 outbreak in northern Italy since early October. Clearly, whether Sardinia is included in the model has little influence on the final result of early warning.


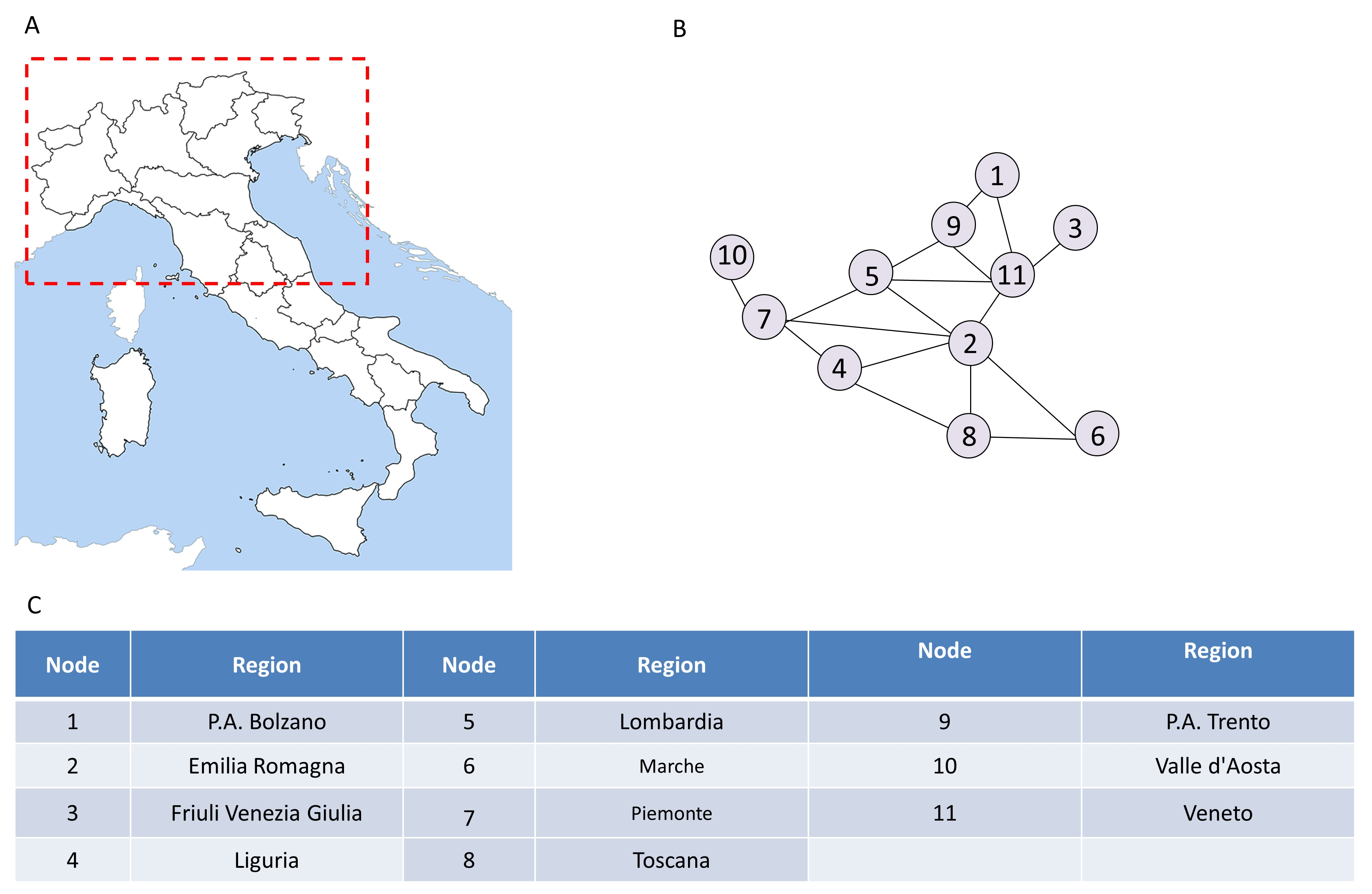


**Figure S2: The region network model of northern Italy.** (A) Map of northern Italy. (B) A 11-node network model is constructed based on the geographic information and adjacent relationships of 11 regions/autonomous provinces in northern Italy. (C) A detailed list of correspondences between regions/autonomous provinces and nodes.


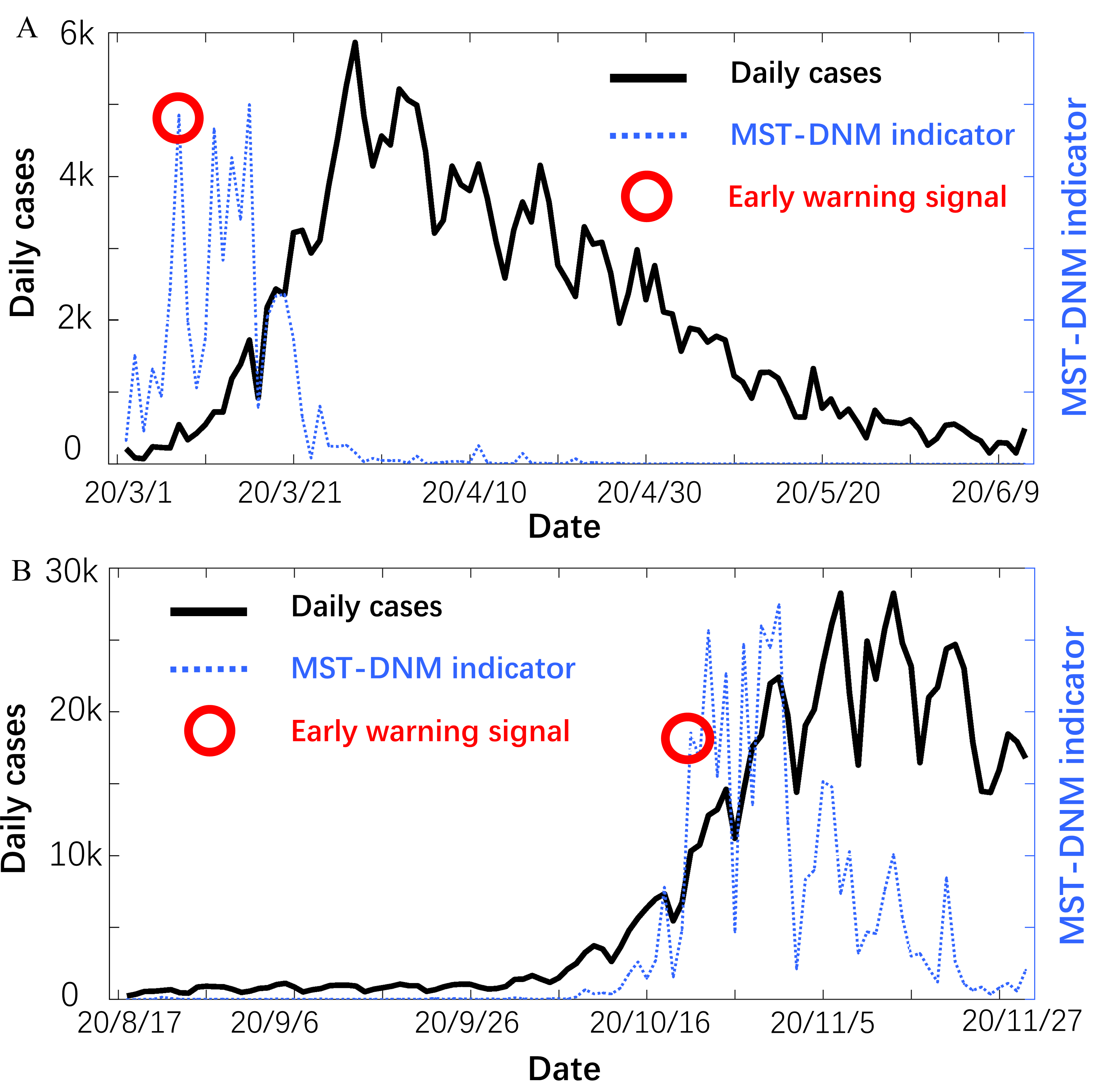


**Figure S3: Forecasting of COVID-19 outbreaks in northern Italy.** In each subgraph, the left y-axis is the number of daily cases in in all regions of northern Italy and the right y-axis is the MST-DNM indicator of corresponding date. (A) Forecasting and warning of the first wave of COVID-19 outbreak in northern Italy. (B) Forecasting and warning of the second wave of COVID-19 outbreak in northern Italy since early October. Clearly, when the actual number of confirmed cases has not increased significantly, significant changes in the indications have been detected, indicating the presence of pre-outbreak signals.
